# Bibliographical Notices

**Published:** 1846-10

**Authors:** 


### BIBLIOGRAPHICAL NOTICES.

*Terminologisches Wörterbuch der Medicinischen Wissenchaften.* Von Dr. Friederich Julius Siebenhaar, Koniglichem Bezirksarzte and praktischem Arzte in Dresden, u. v. w. 8vo. s. 722. Dresden und Leipzig. 1842.

*Terminological Dictionary of the Medical Sciences.* By Dr. Frederick Julius Siebenhaar, &c. &c. 8vo. pp. 722. Dresden & Leipsic. 1842.

*A Pentaglot Dictionary of the Terms employed in Anatomy, Physiology, Pathology, Practical Medicine, Surgery, Obstetrics, Medical Jurisprudence, Materia Medica, Pharmacy, Medical Zoology, Botany and Chemistry.* In two Parts: Part 1. With the leading term in French, followed by the Synonymes in the Greek, Latin, German and English; explanations in English; and copious illustrations in the different languages. Part 2. A German-English-French Dictionary, comprehending the scientific German Terms of the preceding part. By Shirley Palmer, M. D., of Tamworth and Birmingham. 8vo. pp. 656. London: 1845.

*A Universal and Critical Dictionary of the English Language; to which are added Walker's Key to the Pronunciation of Classical and Scriptural Proper Names, much enlarged and improved; and a Pronouncing Vocabulary of Modern Geographical Names.* By Joseph E. Worcester. Royal 8vo. pp. 956. Boston: 1846.

To the student of any science, nothing is more important than a dictionary of terms, provided the explanations be sufficiently full to convey precise information. It may be, that he is merely desirous of learning the general acceptation of a word, which may be a source of difficulty to him, and under such circumstances a brief explanation may be sufficient; but it as often happens, perhaps, that he wishes to know, in epitome, the general and special relations which the term has to the science ; and in such case a mere Glossary does not adequately supply his wants. But dictionaries are not alone necessary to the tyro. In the perpetual modifications and improvements, that are taking place in science, its language becomes enriched by new terms, or by modifications of the old, and hence a work which brings from time to time these terms up to the existing state of science becomes almost indispensable. In the last edition of his Medical Dictionary, Dr. Dunglison states, that he had added nearly two thousand five hundred subjects and terms not contained in the previous edition published a year or two before,—many of these having been introduced into medical terminology in consequence of the progress of the science, and others having escaped him on previous revisions; and Mr. Worcester affirms, that in his Dictionary of the English language now before us, to which he has added scientific terms, *twenty-seven thousand words* are to be found not contained in Todd's edition of Johnson's Dictionary.

The Dictionaries, whose titles are at the head of this article, are essentially Dictionaries of Terms, Glossaries or Vocabularies. The German Dictionary of Dr. Siebenhaar is much less full than that of Dr. Palmer. The plan pursued by Dr. S. is to give the quantity, so as to regulate the pronunciation and the etymology ;—classing all terms together that are formed from the same radical word, as in the following example.

"ADEPS, ĭpis. gen. com. Fat, grease, same meaning as Sevum. Adj. ADIPĀTUS and ADIPŌSUS, containing fat ; e. g. *Panniculus adiposus*, the fatty membrane—ADIPOCĒRA (from adeps and cera, wax.) Adipocire; Germ. Fettwachs—ADIPOCERŌMA (from adeps and κηρος, wax.) The formation of adipocire; of the same signification with cholestearinoma." p. 10.

To the work are prefixed some interesting philological remarks on the proper rules for forming scientific terms ; with the meaning of different prefixes and suffixes from the Greek, the language which is alone used for the formation of such terms; or rather, which ought to be alone used, for we often meet with hybrid formations, partly Greek and partly Latin, which ought to be discarded, and against which the author very properly, raises his voice,—instancing especially *Abdominoscopia*, *Coxarthrocace*, *Duodenopyra*, *Ovariophthisis*, *Pelvimetria*, *Rectostenosis*, *Sacrocoxalgia*, &c., p. xvi. In the body of the work he properly objects to such terms as the following.

"DESCEMETITIS, formed entirely against all philological usage, from Descemet, the name of a French ophthalmic surgeon; and the termination *itis*, which denotes inflammation. Inflammation of Descemet's membrane of the eye. (Properly inflammation of Descemet!") p. 56.

Such hybrid and often unmeaning terms we are doomed to meet with occasionally, and at times from those who ought to know better. The author has heard *Dyspeptitis* used by an intelligent physician to signify dyspepsia connected with chronic inflammation of the lining membrane of the stomach;—the term really importing "inflammation of dyspepsia;" and *Rachialgitis* is constantly employed by some for spinal irritation;—a term, which—if it means any thing—signifies "inflammation of pain in the spine"!

The Dictionary of Dr. Siebenhaar is so distinguished for philological accuracy, that we are satisfied that the few errors in it, even of quantity—such as *Conĭum* and *Perfŏrans*—are entirely typographical. We have found it of essential service, inasmuch as it contains most of the modern technical terms connected with medicine that have originated in Germany. Prefixed is the following brief tabular chronological view of the History of Medicine, which is inserted by the author for the better "understanding of some of the proper names used in the Dictionary." As we have often found the want of such a table of reference, we translate it, notwithstanding its imperfection, and its too German character.

| | | |
|---|---|---|
| 1672 | B. C. | First mention of physicians in Egypt. |
| 1250 | - - | Æsculapius, (1370 translated amongst the gods; 690 worshipped at Rome.) |
| 584 | - - | Nebrus and Chrysus, Asclepiades. |
| 580 | - - | The philosopher Pythagoras born. |
| 460 | - - | Hippocrates, (II.) son of Heraclides, born. |
| 430 | - - | Plague in Athens, and birth of Plato. |
| 384 | - - | Aristotle born, (philosopher and natural historian.) |

| | | |
|---|---|---|
| 377 | B. C. | Death of Hippocrates, (according to others, 370.) |
| 371 | - - | Theophrastus born, (botanist.) |
| 374 | - - | Thessalus, founder of the dogmatic school. |
| 348 | - - | Death of Plato, (philosopher.) |
| 340 | - - | Zeno of Kittium, born (philosopher.) |
| 322 | - - | Aristotle born. |
| 307 | - - | Herophilus of Chalcedon (Anatomy of the Human Body.) |
| 304 | - - | Erasistratus, (anatomist.) |
| 290 | - - | Death of Theophrastus; Philenus, founder of the empirical school. |
| 279 | - - | Eudemus (celebrated anatomist); Serapion (empiric.) |
| 276 | - - | Glaucias, the empiric; Ammonius, the lithotomist. |
| 261 | - - | Death of Zeno. |
| 219 | - - | Archagathus, the Greek physician, goes to Rome. |
| 138 | - - | Nicandar teaches the agents against poisons. |
| 100 | - - | Asclepiades goes to Rome. |
| 63 | - - | Themison of Laodicea, (first methodist.) |
| 21 | - - | Antonius Musa, first (?) " water doctor." |
| 3—5 | A. C. | Cornelius Celsus, (celebrated Roman physician, and encyclopædist.) |
| 23 | - - | Pliny, the naturalist, born. |
| 54 | - - | Dioscoridės of Anazarba, botanist. |
| 54 | - - | Establishment of body and state physicians, (the first, Andromachus, inventor of the Theriac,) or archiatria: Caius and Euelpides, occulists. |
| 68 | - - | Æthenæus of Attalia, founder of the pneumatic from the dogmatic school. |
| 79 | - - | Pliny died. |
| 81 | - - | Aretæus, uniter of the pneumatic and eclectic systems. Marinus, restorer of the anatomy of the human body. |
| 97 | - - | Archigenes, founder of the eclectic school; Rufus of Ephesus; Cassius, the Iatrosophist; Soranus, teacher of the treatment of fractures; Heliodorus, the surgeon. |
| 117 | - - | Moschion, teacher of obstetrics and the diseases of children. |
| 131 | - - | Galen born at Pergamus. |
| 138 | - - | Marcellus of Sidæ, observer of lycanthropia. |
| 165 | - - | Galen goes to Rome. |
| 203 | - - | Galen dies. |
| 230 | - - | Cœlius Aurelianus, the methodist. |
| 237 | - - | Serenus Sammonicus II. (I. 222) dietetist. |
| 296 | - - | Diocletian's edict against alchemy. |
| 357 | - - | Constantine's edict against magic (repeated 367.) |
| 360 | - - | Oribasius, compiler of the earlier writers. |
| 541 | - - | General plague. |
| 543 | - - | Benedict of Nursia founds the cloister of Monte Cassino, where first in the middle ages practical and theoretical medicine was carried on; Aetius, of Amida, compiler and galenist; Alexander, of Tralles, self-thinker. |
| 565 | - - | Small pox in France. |
| 572 | - - | Do. in Arabia. |
| 622 | - - | Aharun, priest of Alexandria, first Arabian writer in the department of medicine. |
| 634 | - - | Paulus of Ægina, celebrated surgeon and obstetrician. |
| 702 | - - | Geber, the Arabian, born. |
| 772 | - - | George Bakhtischwah, of the Nestorian-Syrian family of physicians. |

804 A. C. Jahia ebn Masawaih (Mesue I.) and Hhonain eb Jzdak, celebrated Arabian physicians.
826 - - Jahia ebn Serapion, the Syrian, compiler of the older Greek physicians.
880 - - Jacob Alkindi died.
923 - - Mohammed Arrasi (Rhazes) died.
936 - - Theophanes (Nonus) or Michael Psellus collects extracts from the older Greek physicians. The Hippiatrika was collected and probably translated into Latin under the name of Vegetius, as *Mulo-Medicina.*
978 - - Ebn Sira (Avicenna) born, (died 1036.)
984 - - Commencement of travels to Salerno for medical treatment.
1017 - - Mesue, the younger, died.
1087 - - Constantin the African, (from Carthage,) translator of the Arabian physicians into Latin, died at Salerno.
1098 - - Hildegard, abbess of Bingen, authoress of a *Materia Medica,* born (died 1180;) Regimen Sanitatis Salernitanum.
1110 - - Nicolaus Præpositus, celebrated physician at Salerno.
1143 - - Roger's Medical Laws for Salerno.
1150 - - The patriarch Lucas, of Constantinople, forbids the ecclesiastics to practice medicine. Matthæus Platearius, of Salerno.
1162 - - The oldest English edict against stews.
1179 - - Ebn Zohar died.
1187 - - Gerard of Cremona died at Toledo; translator of the Arabian physicians.
1199 - - Hugo Physicus, teacher of medicine at Paris.
1206 - - Ebn Roschd died.
1209 - - The physical writings of Aristotle prohibited at Paris.
1214 - - Roger Bacon born (died 1295.)
1220 - - Medical Faculty of Montpellier.
1238 - - Frederick II. Medical Laws for Salerno and Naples.
1243 - - Medical school at Damascus.
1248 - - Ebn Berthar died.
1250 - - Scurvy in the army of Louis IX.
1271 - - College of Surgeons of Paris.
1282 - - Albert of Bollstädt (Albertus Magnus) died.
1285 - - Bernard Gordon, of Montpellier; Arnold, of Villanova, professor at Barcelona.
1287 - - Traces of Plica Polonica.
1295 - - Langfranchi of Milan, celebrated surgeon, goes to Paris.
1305 - - Bernard Gordon writes his Compendium of Medicine.
1315 - - Mondini's first public dissections (died 1327.)
1317 - - Matthæus Sylvaticus writes his Medical Pandects.
1348 - - Black death.
1349 - - Jacob de Dondi, Medical Manual.
1363 - - Guy de Chauliac writes his surgical manual.
1374 - - Epidemic St. Vitus's dance on the Rhine.
1376 - - Permission given at Montpellier to dissect.
1406 - - Privilege to the Bathers by the Emperor Wenceslaus.
1409 - - Establishment of the Löwenapotheke at Leipsic.
1485 - - English sweating sickness, (repeated 1506, 1517, 1520, 1528, 1551.)
1488 - - The first formulary (*Receptirkunst*) at Florence.
1491 - - First wood cuts of plants and anatomical objects.
1493 - - Appearance of the venereal disease in France, Italy and Germany. Since 1495, works thereon.

1519 A. C. First mention of guaiac wood.
1520 - - First appearance of gonorrhœa.
1535 - - Symphorian Champier died, (born 1472,) the first who compared the fundamental principles of Greek and Arabian medicine.
1541 - - Chr. Philippus Aureolus Theophrastus Paracelsus Bombastus von Hohenheim, (born 1493 at Einsiedlen,) died at Salzburg.
1545 - - Separation of the surgeons and bathers at Paris by William Vavasseur; botanic garden at Padua.
1552 - - Bartholomæus Eustachi's (died 1574) anatomical plates; anatomical theatre at Pisa; book on midwifery by Eucharius Rösslin.
1553 - - Michael Servetus (born 1507) teaches the lesser circulation of the blood through the lungs, and is burnt at Geneva; Hieronymus Fracastori, the elucidator of the doctrine of critical days, died, (born 1483.)
1562 - - Gabriel Fallopia (born 1523) died.
1564 - - Andreas Vesalius, (born 1515,) the critic of Galen, and the anatomist, died.
1565 - - Conrad Gesner, of Zürich, (born 1516,) the great natural historian, died.
1571 - - Cæsalpini, (died 1603,) the celebrated classifier of plants, teaches the circulation of the blood; colic of Poitou.
1582 - - The creeping sickness appears at Lüneburg.
1590 - - The celebrated surgeon Ambrose Paré died at Paris.
1617 - - The semeiologist and botanist, Prosper Alpini, died, (born 1553.)
1619 - - William Harvey, of Folkstone, in Kent, (died 1657,) teaches publicly the circulation of the blood.
1626 - - The true leprosy disappears in France.
1630 - - Caspar Hoffman, professor at Altorf, (died 1642,) opponent of Harvey.
1640 - - Cinchona carried to Europe.
1641 - - Marcellus Malpighi (died 1694) discloses his ideas on the structure of the lungs; Thomas Sydenham (died 1689) begins his observations on epidemics.
1644 - - Van Helmont (born 1577 at Brussels) died, after having set up his spiritual system.
1650 - - René des Cartes of Haye, in Touraine, died at Stockholm.
1656 - - Plague in Italy.
1658 - - Francis de la Boe Sylvius taught the chemical system at Amsterdam.
1665 - - Plague in London.
1672 - - Newton discovers the true nature of colours and the refraction of light.
1679 - - Plague in Germany.
1688 - - The work of Paul Zacchias (physician to the Pope, died 1659) on legal medicine, (Rome 1621,) is known in Germany.
1707 - - George Louis Le Clerc, Count Buffon, the teacher of a new theory of generation, born, (died 1788.)
1708 - - Albert von Haller, (died 1777,) a great anatomist and physiologist, born.
1712 - - Fred. Hoffmann, of Halle, (born 1660, died 1742,) teaches there his mechanico-dynamic system.

1716 A. C. George Ernest Stahl, (born in 1660 at Anspach,) goes as body physician to Berlin from Halle, where he had taught since 1694 his psychical system, (died 1734;) Raymond Vieussens (born 1641) died, after he had made known his observations on the structure of the heart.

1717 - - Lady Mary Somerset Montague suffers her son to be inoculated at Constantinople.

1727 - - J. A. Unzer, physician at Altona, the founder of the nerve theory, born.

1728 - - John Hunter, the celebrated English surgeon, born, (died 1793.)

1730 - - The yellow fever (*vomito prieto*) appears at Carthagena.

1734 - - Anthony Mesmer born at Vienna, (died 1815,) the improver of the magnetic method of cure.

1735 - - John Brown born, the propounder of the excitement theory, (died 1788;) Linnæus teaches the sexual system of plants.

1738 - - Hermann Boerhaave, the overthrower of the chemical school, died.

1743 - - Ant. Laur. Lavoisier (died 1794) born in Paris; the founder of the doctrine of antiphlogistic chemistry.

1745 - - Chr. Gottl. Kratzenstein writes his book on the application of electricity to medicine.

1751 - - Establishment of the Berlin school of midwifery.

1755 - - Samuel Christian Frederick Hahnemann, founder (1790) of the homœopathic method of cure, born at Meissen.

1757 - - Fr. Joseph Galt, the proposer of the craniological theory, born at Tiefenbronn, near Pforzheim, died in 1828 in Paris.

1758 - - Lawrence Heister, (born 1633,) the first German scientific surgeon, died.

1768 - - Edward Jenner, of Berkeley, in England, discovers vaccination.

1774 - - John Baptist Biot, the Parisian natural philosopher, born.

1790 - - The *mal di scherlievo* appears at Fiume, and the scarlet fever in Northern Germany.

1797 - - George Prochaska, professor of physiology at Vienna, proposes his galvanic theory of life.

1799 - - Aloysius Galvani, professor at Bologna, and inventor of the doctrine of electric elasticity, (*spannkraft*,) died, (born 1737.)

1800 - - Appearance of the influenza, or *grippe*, in Europe; of the petechial fever in Genoa, and of the yellow fever in Cadiz.

1802 - - Xavier Bichat, the celebrated Parisian anatomist, died, (born 1771.)

1803 - - Philip Pinel, of Paris, makes known his arrangement of diseases.

1807 - - Ludwig Oken, the natural philosopher, professor at Jena, (since 1833, at Zürich;) Sir Humphry Davy (born 1779, died 1836,) discovers, with the aid of the Voltaic pile, that alkalies and earths, previously regarded as simple bodies, are combinations of a metal with oxygen.

1811 - - Epidemic puerperal fever at Heidelberg; Er. v. Grossi teaches at Munich the different families of diseases, and gives them new names.

1817 - - The cholera orientalis appears in India.

1819 - - R. T. H. Laennec invents the stethoscope and auscultation.

1820 - - The chemical work of Jacob Berzelius, (born 1779, now professor at Upsala,) the discoverer of the dualistic or electro-chemical system, is known in Germany.

1821 A. C. J. P. Frank, (born 1745 in Badeschen,) the classifier of diseases, died.
1830 - - Samuel Thomas Sömmering, the reformer of anatomical terminology, died.
1832 - - George Cuvier, (born 1769 at Mümpelgard,) the comparative anatomist, died.
1833 - - J. Fr. Meckel, anatomist of Halle; and Dupuytren, celebrated French surgeon, died.
1836 - - Chr. William Hufeland, (born 1762 at Langensalza,) the celebrated eclectic, died at Berlin.
1838 - - Fr. J. Broussais, (born 1772,) the propounder of the inflammation theory, died.
1837 - - Giovanni Rasori, the proposer of the doctrine of the contra-stimulus, died at Milan.
1840 - - John Lucas Schönlein, (born 1793 at Bamberg,) the founder of a natural historical pathology, goes as professor from Zürich to Berlin."—p. xxviii.

The Pentaglot Dictionary of Dr. Palmer has been long in progress. Many years ago, he was applied to "by an intelligent and enterprising publisher" of Birmingham to compile for the use of the medical student a Dictionary of French and German scientific terms. The proposition was favourably entertained, and in the summer of 1834 the first part was published; the second appeared in the spring of 1836; and the third and last part of his "protracted labours" was issued in 1845. The toil must, indeed, have been considerable; and we fear the reward not in proportion, for valuable as such a work must be to one who is able to peruse the authors of Continental Europe, these can form but a small proportion of the mass. The fact, too, of the French being taken as the leading language of the work, will be a prohibition to the mere English reader.

"The principle," says Dr. Palmer, "upon which I have been induced to select the French as the leading language of the work has frequently been questioned, and discussed by literary men. My reply is, that it was expressly intended to assist the uninitiated in acquiring a correct knowledge of French and German medical and scientific literature; and as the French is more extensively studied than the German, in this country, and is generally spoken, or read, in all the great medical schools and scientific Institutions of Europe, the expediency of rendering it a French Dictionary is at once obvious. Upon this ground, I am induced to hope that my work may acquire not only a British but an European circulation. The French possesses, moreover, the signal advantage of furnishing a great number of modern scientific terms, which will be in vain sought for in the Latin, and in other European languages." p. vii.

A single extract will show the plan adopted by the author in the body of the work. We take the following at random.

"LYMPHATIQUE, adj.—lymphaticus, L.—lymphatic, containing or relating to *lymph*, LYMPHE, s. f.—lympha, f. L.—die Lymphe, G.—or the *vessels*, by which it is conveyed: an epithet employed in anatomy to designate, 1, those vessels—les *vaisseaux lymphatiques*, F.—vasa *lymphatica*, L.,—die *lymphatischen* Gefässe, G.; or 2, the glands,—*ganglions lymphatiques*, situated in their course; 3, the whole *system of organs*,—*Système ou apparel lymphatique*,—which contributes to the elaboration and transmission of the lymph: in Pathology, a *temperament*,—T. *lymphatique*, wherein the *lymphatic* system predominates. L. de Cotugno: the *fluid* which fills all the cavities of the internal ear." p. 360.

The last one hundred and twenty pages is a German-English-French-Dictionary of the scientific terms contained in the preceding part of the work—the German term coming first.

We can strongly recommend Dr. Palmer's work. We have run over many portions of it, and find it exceedingly accurate. To the reader of French and German works it will be an excellent accompaniment.

We notice the large "Universal and Critical Dictionary of the English language" of Mr. Worcester, because he has introduced into it the terms used in medicine, as well as in other departments of science. "The technical and scientific terms," he observes, "have generally been taken from scientific works, or from dictionaries of the various arts and sciences; as Brande's "Dictionary of Science, Literature and Art;" Ure's "Dictionary of Arts, Manufactures, and Mines;" Crabb's "Technological Dictionary;" Falconer's "Marine Dictionary;" Dunglison's "Medical Dictionary;" Bouvier's "Law Dictionary;" Loudon's "Encyclopædias;" the "Penny Encyclopædia," and many other dictionaries of the different arts and sciences, and various encyclopædias, the titles of which are to be found in the catalogue of works of this kind, in the Introduction of this volume." p. v.

We would merely remark of one of these authorities—Crabb—that he is most unaccountably inaccurate in many of the acceptations which he gives to terms even in common use. He is evidently, indeed, ignorant of their meaning; and hence must lead into error those who place reliance upon him. We have often had occasion to consult the work, and have become disgusted with its shallow and idle pretensions. He has evidently led Mr. Worcester into error, and in a department where the latter had another work, which he gives amongst his authorities, to consult: Thus—*Fluor albus* is defined by Mr. Worcester,—"a diseased state of the menses!" and the authority given for it is Crabb. A greater amount of ignorance could not be exhibited in so small a compass; yet it is but one specimen from a multitude with which the "Technological Dictionary" abounds.

Some idea of the labour bestowed by Mr. Worcester upon this Dictionary, may be formed from the fact already stated, that he has added *twenty-seven thousand* words that are not to be found in Todd's edition of Johnson's Dictionary. His "Comprehensive pronouncing and explanatory Dictionary of the English language" is doubtless known to most of our readers, and as highly valued as it is extensively known. It has been the comfort of the more advanced student as well as of the tyro; but the present extensive work is calculated for a wider sphere of usefulness, and should be in the library of every one who desires to have at hand a dictionary, that can give him information in regard to most of the words which he may meet with in his varied readings, and that can at the same time instruct him in regard to their proper orthoepy.

---

*On Man's power over himself to prevent or control Insanity.* By the Rev. John Barlow, M. A., of Trinity College, Cambridge. 16mo. pp. 68. London, Wm. Pickering: 1843.

The volume whose title we have given above, as we are informed by its author, contains the substance of a communication made to the Members of the Royal Institution of Great Britain, and was published at the requisition of the President of that body. Though given to the public under such high sanction, it contains and is written with the object of enforcing views which are somewhat at variance with those generally received by physicians who have given the most attention to the study of Insanity. Its recent publication in this country gives us the opportunity of offering a comment or two on some of its doctrines.

In order to support his theory of Insanity, our author assumes the existence of two forces which he says manifest themselves in the phenomena of man's nature. "The vital force, by virtue of which he is an animal; and the intellectual force, by virtue of which he is something more." It is the province of the intellectual force to regulate the operations of the mind, and the motions of the vital force, and insanity is the consequence of a defect in the exercise of this regulating power. "He who has given a proper direction to his intellectual force, and thus attained an early command over the bodily organ, by habituating it to processes of calm reasoning, remains sane amid all the vagaries of sense, while he who has been the slave, rather than the master of his animal nature, listens to its dictates without question even when distorted by disease—and is mad." p. 12.

The author considers mental disorders to be of two kinds: the first caused by morbid affections of the nervous system and brain, including delirium, delusions as to sight, sounds, &c., and loss of memory—the second arising from morbid affections of the intellectual force. Affections of the nervous system and brain never of themselves cause insanity, unless of the greatest severity. "Nothing but an extent of disease which destroys at once all possibility of reasoning, by annihilating or entirely changing the structure of the organ, can make a man necessarily mad. In all other cases, the being sane or otherwise, notwithstanding considerable disease of the brain, depends on the individual himself." p. 12.

Thus, it will be seen, a distinction is made between mental derangement, caused by actual disease of the nervous system, constituting the first, and insanity, strictly speaking, resulting from the want of a proper exercise of the intellectual force, composing the second class of mental diseases.

This fabric is so completely demolished by the experience of every one who has seen any thing of mental disorders, that it is not needful for us to say one word against it. Probably not one case in every hundred of mental derangement would come within the bounds of either of the above classes.

An extract or two will show more fully the writer's views of the nature of what he calls insanity:

When once the intelligent will has lent its force to the blind impulses of the body, whether diseased or in health, it becomes only a question of time whether the individual is to be called insane, and placed under restraint or not. The man who recovers quickly from his madness is called a sane man, though during the preceding minutes or hours, he may have exhibited the flushed face, the rapid and violent gestures and language, and the unreasonable conclusions of a manaic; but strange to say, if this be very frequent, he is excused and considered innocent of the crime he perpetrates, exactly because he has committed the greatest of all crimes, by delivering over his god-like intellect to be the sport of that brute nature which it ought to regulate." p. 39.

"Should my position, that the difference between sanity and insanity consists in the degree of self-control exercised, appear paradoxical to any one, let him note for a moment the thoughts that pass in his own mind and the feelings that agitate him, and he would find that were they all expressed or indulged, they would be as wild, and perhaps as frightful in their consequences, as those of any madman. But the man of strong mind represses them, and seeks fresh impressions from without if he finds that aid is needful; the man of weak mind yields to them and then he is insane." p. 45.

"The result, then, of the whole inquiry appears to be, that man being a compound of two natures, mental derangement is of two kinds—in the one kind, structural disease deadens and distorts the perceptions, and if this extends itself to the organs of all the faculties, the intellectual force having no longer the means of external action, the individual remains to all appearance a helpless machine."

"But in the other case no structural disease exists in the first instance, and the inefficiency or misdirection of the intellectual force is the sole cause of derangement, sometimes as I have already noticed, continuing to the last without effecting the bodily organs." p. 489.

Setting aside the argument which may be derived from the hypothetical character of this intellectual course and its consequent doubtful ability to influence, in any manner, the mental operations, it will only be necessary, in order to show the fallacy of such a view of the nature of insanity, to adduce the conviction of Esquirol and others, that the will itself is liable to the same perversions and disturbances as the other faculties, and to advert to the numerous instances that have been related by the French and German authors in illustration of a form of insanity, in which there is a blind desire, sometimes insurmountable, to commit murder—the will alone appearing to be affected, while the reasoning powers and moral feelings are in a sound state. In the case which has been so often quoted of the domestic in Baron Humboldt's family, and others of a similar kind, so far from the animal nature being at fault, and the "intellectual force" or "intelligent will" preserving the girl from the commission of a great crime, according to the theory of this work, we find a mental condition nearly the reverse; the will being strongly impelled to the act, while the natural feelings are engaged in an arduous conflict, and at last succeeding in preventing its accomplishment. Our author, however, causes this difficulty to vanish by the exercise of his ingenuity in excluding these cases from the title of insanity, and considering them only as mental derangement. The intellectual force kindly interferes and shows the sufferer the enormity of his desires, and preserves him sane amid all the vagaries of his senses. He is only mentally deranged as long as he resists impulses at which his nature revolts; he becomes insane when he delivers over his god-like intellect to be the sport of his natural propensities.

It is well known to all acquainted with the features of this disease, that many insane persons possess the power of controlling completely all evidence of their malady, sometimes for long periods, while at the same time they may be entertaining feelings inconsistent with a sound mind. According to the author's ideas these persons must in future only be considered suffering from an aberration of the "vital force," they are not insane because they are able to control themselves, while any man who gives way to a fit of sudden anger, and commits an action which the next moment he may repent, must for the time being be called insane.

The above are but a part of the inconsistences and contradictions into which the doctrines of the work lead, when applied to the interpretation of facts of daily observation. We trust that their being thus hastily pointed out, may be sufficient, in some measure, to guard against the inconsiderate adoption of views which are well set forth in the forcible manner in which a few facts are made to support them, and what is of serious consequence, views which are strongly opposed to an enlightened jurisprudence of insanity.

---

*Experimental Researches on the Post-Mortem Contractility of the Muscles, with Observations on the Reflex-Theory.* By Bennet Dowler, M. D. Pamphlet, pp. 39.

Our pages have in a former number contained some account of Dr. Dowler's experiments in reference to *post-mortem caloricity;* since then, the profession has been made acquainted through other publications with his observations on *post-mortem contractility*, which are little if at all less curious and suggestive than those on the former subject.

In the first of the pamphlets now before us, we have many experiments detailed, going to prove that muscular contraction will occur, responsive to the simplest mechanical stimulus, many hours after death, even although the part be entirely severed from all connection with the brain and spinal marrow. The phenomena observed during these experiments, in Dr. Dowler's opinion entirely upset the doctrine of reflex nervous action.

The author objects to the means employed by the advocates of the reflex-theory to establish their doctrine, on various grounds, as irrational and inappropriate.

In the first place, the phenomena on which they have relied to prove their theory have been exhibited only in cases of vivisection; which, beside the inhumanity of such proceedings, necessarily lead to confusion or erroneous conclusions in consequence of the unnatural condition of all the functions of the animal under the circumstances.

Secondly: he regards the conclusions drawn from what is observed in the experiments by vivisection of the lower order of animals, as inapplicable to the higher organization which exists in man. "It should never be forgotten," he observes, "that experiments on the inferior animals, as frogs and turtles, are inconclusive in establishing the complicated physiology of man. An earth-worm may be cut into several pieces, each of which becomes a perfect animal. The more the *hydræ* are subdivided, the more is their number increased without loss of vitality. Could we deduce a vital or reflex theory from such experiments applicable to man? Were capital punishment changed from hanging to vivisection of the spinal marrow, this would but poorly illustrate the physiology of a healthy man, or the pathology of a sick one; yet one such experiment would be worth a thousand upon frogs."

Numerous experiments performed on the bodies of persons dead from a few minutes to several hours, mostly subjects of yellow fever, are detailed, in which the muscles of the extremities, when struck with a cane, billet of wood, the hand, or the side of a hatchet, contracted with sufficient force to move a weight of several pounds. Two or three of these experiments, selected at random, will serve to show the character of the evidence on which Dr. D. bases his conclusions.

"Case 1.—O. D., an Englishman, aged 26. From fifteen minutes to several hours after death, raised his forearm to the perpendicular as often as its flexors were struck; the motion was slow, the ratio of contraction and relaxation appeared uniform, occupying about half a minute."

"Case 2.—E. M., an Englishman, aged 37. At thirty minutes after death, afforded similar results. The experiments were often repeated, at prolonged periods; the contractions and relaxations were supposed to occupy about thirty seconds each. The flexion ceased at the perpendicular in every instance. When the experiments ceased, the muscular force continued at its maximum, having lost nothing."

"Case 20. *Remittent.*—Mr. S., aged 45. Dead two hours. Legs becoming rigid; struck the flexors of the arm with the inferior edge of my hand; the cadaver raised his arm with a regular, slow movement, placing his hand upon his breast: as soon as the muscles relaxed, he carried his arm back, extending the same. The experiment was repeated three or four times, when the arm fell back exhausted. The blows were now made with a piece of wood. The biceps gathered up into a lump, at the place where the blow was given, but failed to move the fore-arm."

"*If several blows on the same spot follow each other rapidly, there is but one contraction, but they exhaust the contractile function more than a single blow; if the force be greatly augmented, the contractility may be killed, almost immediately in the muscle struck, without impairing the action of any other part.*"

Dr. Dowler regards these and other like experiments, which he has detailed, as conclusive proofs against the *reflex-theory.* "Why," he asks, "should a very simple matter be thus complicated by galvanic experiments, dissections, and reflex logic, seeing that a blow direct with the hand will produce results more definite, more like the acts of vitality and volition? If an arm, from six to fourteen hours after death, hours after dissection of the body, and after being severed from the trunk at the shoulder joint, contract with the utmost precision, how can gentlemen say 'that the anterior columns of the spinal cord possess an exclusive motor function'? Whatever agency these roots may have when galvanized, they are incomparably inferior to the motor power of the muscle when excited by the hand, the hatchet, or a cane."

